# Comparative Analysis of Codon Usage Bias and Codon Context Patterns between Dipteran and Hymenopteran Sequenced Genomes

**DOI:** 10.1371/journal.pone.0043111

**Published:** 2012-08-17

**Authors:** Susanta K. Behura, David W. Severson

**Affiliations:** Eck Institute for Global Health, Department of Biological Sciences. University of Notre Dame, Notre Dame, Indiana, United States of America; George Washington University, United States of America

## Abstract

**Background:**

Codon bias is a phenomenon of non-uniform usage of codons whereas codon context generally refers to sequential pair of codons in a gene. Although genome sequencing of multiple species of dipteran and hymenopteran insects have been completed only a few of these species have been analyzed for codon usage bias.

**Methods and Principal Findings:**

Here, we use bioinformatics approaches to analyze codon usage bias and codon context patterns in a genome-wide manner among 15 dipteran and 7 hymenopteran insect species. Results show that GAA is the most frequent codon in the dipteran species whereas GAG is the most frequent codon in the hymenopteran species. Data reveals that codons ending with C or G are frequently used in the dipteran genomes whereas codons ending with A or T are frequently used in the hymenopteran genomes. Synonymous codon usage orders (SCUO) vary within genomes in a pattern that seems to be distinct for each species. Based on comparison of 30 one-to-one orthologous genes among 17 species, the fruit fly *Drosophila willistoni* shows the least codon usage bias whereas the honey bee (*Apis mellifera*) shows the highest bias. Analysis of codon context patterns of these insects shows that specific codons are frequently used as the 3′- and 5′-context of start and stop codons, respectively.

**Conclusions:**

Codon bias pattern is distinct between dipteran and hymenopteran insects. While codon bias is favored by high GC content of dipteran genomes, high AT content of genes favors biased usage of synonymous codons in the hymenopteran insects. Also, codon context patterns vary among these species largely according to their phylogeny.

## Introduction

Codon usage bias (or simply codon bias) is a phenomenon of non-uniform usage of codons during translation of genes to proteins. Specific codons are used more often than alternate synonymous codons in genes where such bias exists. Such codons are often referred to as optimized codons or preferred codons. The extent of codon bias is manifested by balance between mutation and translational selection [1], [2], [3], [4]. The bias is most prominent in certain types of genes, generally in highly expressed genes. Variation of codon optimization among genes provides differential efficiency as well as accuracy in the translation of genes [5], [6], [7]. The selection associated with translational efficiency/accuracy is often termed as ‘translation selection’.

The study of codon bias is gaining renewed attention with the advent of whole genome sequencing of numerous organisms [8]. Molecular evolutionary investigations suggest that codon usage bias varies both within and between genomes and may have significant relevance to understanding genome evolution among related species [9]. In the post-genomic era, studies on codon bias are now being directed towards analyzing the whole coding genome rather than specific genes or sets of genes, to better understand translational selection of genomes [8]. Various factors such as expression level, gene length, composition bias (%G+C content and GC skew), recombination rates and RNA stability, among others, are known to influence codon bias [4], [10], [11], [12]. Genome-wide investigations of codon bias patterns, their causes and consequences, and identification of selective forces that shape their evolution are of significant importance to studies of genome biology. Apart from non-random usages of synonymous codons, the codon context sequences are also important features of genes as these sequences correspond to the A and P sites of the ribosome during translation. The A site binds to an aminoacyled tRNA and the P site binds to a peptidyl-tRNA (a tRNA bound to the peptide being synthesized). Thus, at any given time, a codon context occupies these two sites of an active ribosome and hence optimization of codon context sequence may have profound effect on the translation selection of genes [13].

Analyses of codon usage patterns in insects are of particular interest for several reasons. The exceptional diversity of insects within the animal kingdom makes insects suitable for studying codon bias patterns at variable evolutionary time scales. Moreover, the recent expansion of genome sequencing of different insect species complexes (such as the twelve *Drosophila* species, several species of ants, honey bee, wasp and multiple mosquitoes) also provides an exceptional opportunity for studying the evolution of codon bias within specific families and genera. In this context, one of the major objectives of this work is to understand the patterns of codon usage bias among dipteran and hymenopteran sequenced genomes. The Diptera and Hymenoptera orders include the majority of insect genomes that have been sequenced so far. Although studies have been conducted on codon usage bias within the *Drosophila* genus or between a few other insect species [14], [15], [16], a comprehensive analysis of codon bias patterns between dipteran and hymenopteran sequenced genomes is lacking. The purpose of this study is to perform comparative analyses of codon usage bias and codon contexts pattern among the sequenced genomes of these two orders. We analyze whole genome sequences of 22 insect species belonging to the two orders in this investigation. Our results provide useful insights on patterns of codon usage bias and codon context sequences that facilitate better understanding of structure and evolution of coding sequences of these insects.

## Results

### Variation of Relative Synonymous Codon Usages between Dipteran and Hymenopteran Species

**Figure 1:**
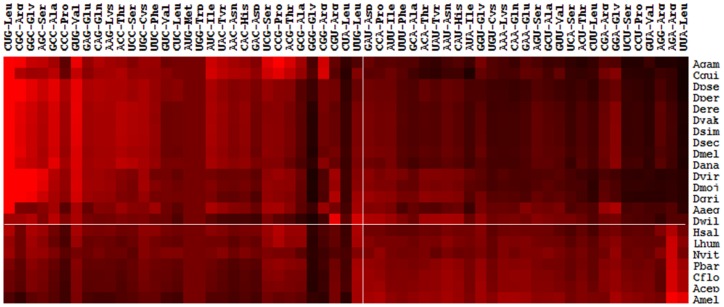
Hierarchal clustering of RSCU values of each codon among the 22 insect species. Each square on the self-organizing map represents the RSCU value of a codon (shown in columns) corresponding to the species (shown in rows). The color coding varies from black to red with low to high values of the RSCU respectively. The top-left quadrant shows the cluster of C or G ending codons that are frequently used in dipteran species relative to the hymenopteran species. The bottom-right quadrant represents the cluster of A or U ending codons that are frequently used in hymenopteran species relative to the dipteran species.

To understand the pattern of non-random usage of synonymous codons in these insects, relative synonymous codon usage (RSCU) of individual codons were compared between the two groups of species. RSCU>1 represents codons that are used more frequently than expected whereas RSCU<1 represents codons which are used less frequently than expected. The expected number is the total number codons divided by the number of synonymous codons of each amino acid. The number of frequently used codons and rarely used codons varies among the species (Table S1). It was found that a total of 29 codons are used in significantly biased (p<0.05) manner between dipteran and hymenopteran insects. These codons are used either as frequent codons in dipteran species but as rare codons in hymenopteran species or *vice versa* (Table 1). Of these, three codons (CGC/Arg, UCC/Ser and ACC/Thr) are consistently used as frequent codons in each dipteran species but as rare codons in each of the hymenopteran species. Similarly, the codon AGA (Arg) is used a frequent codon in each of the hymenopteran species but as rare codon in each of the dipteran insects. Furthermore, it was also found that specific codons are frequently used in each species of both the groups (Table S1). Based on hierarchical clustering of relative synonymous codon usage indices (Figure 1), it was observed that codons ending with C or G are used frequently in dipteran genomes whereas codons ending with A or U are used frequently in the hymenopteran genomes. We didn’t find such a distinction in the sequence patterns of the rare codons between diptera and hymenoptera. In fact, specific codons (such as GGG and CUA) are identified that are commonly avoided in each species of the two orders (Table S1).

**Table 1:** Codons that are used more frequently than expected (RSCU >1) among the dipteran and hymenopteran insects.

Codon	Diptera	Hymenoptera	Significance
**CGC - Arg**	15	0	5.86E-06
**UCC - Ser**	15	0	5.86E-06
**ACC - Thr**	15	0	5.86E-06
**GCC - Ala**	15	1	9.38E-05
**CUG - Leu**	15	4	0.022727
**GAG - Glu**	14	1	0.000622
**AAG - Lys**	14	1	0.000622
**CAG - Gln**	14	2	0.004316
**AUC - Ile**	13	2	0.013551
**UUC - Phe**	13	2	0.013551
**CCC - Pro**	12	0	0.000704
**AAC - Asn**	11	0	0.00387
**ACA - Thr**	4	6	0.020124
**AAU - Asn**	4	7	0.00387
**GGU - Gly**	4	7	0.00387
**UUU - Phe**	2	5	0.013551
**GCU - Ala**	1	5	0.004316
**CGA - Arg**	1	5	0.004316
**CAA - Gln**	1	5	0.004316
**AGU - Ser**	1	5	0.004316
**GAA - Glu**	1	6	0.000622
**AAA - Lys**	1	6	0.000622
**UCU - Ser**	0	3	0.022727
**GUA - Val**	0	3	0.022727
**GCA - Ala**	0	4	0.004785
**AUA - Ile**	0	4	0.004785
**ACU - Thr**	0	4	0.004785
**UUA - Leu**	0	6	9.38E-05
**AGA - Arg**	0	7	5.86E-06

The number of species (out of 15 Diptera and 7 Hymenoptera) in which the codon is frequently used (RSCU >1) is shown. Also shown are Chi square significance levels for the number of species where the codon is frequently used (RSCU >1) compared to the number of species where the same codon is rarely used (RSCU <1) between the two species groups.

### Effect of base Composition on Observed Codon Bias

Due to the difference in mutational bias, the GC percentage among different species varies greatly, even for the species from the same order. It has been shown from earlier studies that genes in hymenopteran genomes are generally localized in GC-poor regions whereas such bias is not evident among dipteran genomes [16]. To determine if GC bias among dipteran and hymenopteran species has an association with codon bias, we resorted to the non-directional codon bias measure ‘effective number of codons (ENC)’ which is known to be dependent upon nucleotide compositions of genes [17]. We adopted a scatter plot method [14] of comparison between ENC and codon adaptation index (CAI) to determine differences between nucleotide composition and codon selection in each species. CAI is a directional measure of codon usage bias similar to relative codon bias score [18] unlike ENC. Thus comparison between CAI and ENC provides a good qualitative assessment between the nucleotide composition and codon bias selection [14]. ENC values vary from 20 to 61 wherein ENC = 20 means that only one codon is used for each amino acid (extreme codon bias) and ENC = 61 means all codons are equally likely to code the amino acids (no biased usage of codons). Thus ENC values less than 61 suggest that the genes are associated with biased usage of codons the extent of which increases as ENC approaches to 20. When the ENC values were plotted against CAI values (Figure S1), negative correlation (Pearson correlation) was obtained between the two in each species. The correlation values ranged from −0.5 to −0.8 among the dipteran insects but remained within −0.2 to −0.4 among the hymenopteran species. About 82–96% of genes showed increase in CAI values with decrease in ENC values in dipteran insects whereas 52–72% of genes showed this trend between ENC and CAI in the hymenopteran insects. Apart from comparing CAI with ENC in individual genomes, a common gene set (n = 698) identified as single copy orthlogous genes across the dipteran and hymenopteran sequenced genomes, were also investigated. These common genes also showed similar correlation between CAI and ENC between dipteran and hymenopteran genome (Figure S1) indicating that codon usage bias of genes of these species have very distinct relationships with nucleotide composition of coding sequences.

To further test the above result, we performed Poisson regression of base compositions of third position of codons (silent position of codons) as predictor variable of ENC variation. The generalized linear model ENC ∼ A3s + G3s + C3s + T3s was fitted with the observed data of ENC and the individual base frequencies of the third positions. The estimated coefficients of the regression analyses are shown in Table 2. They represent variation of ENC with unit change in the nucleotide frequency at the third position. These results show that effective number of codons of genes in hymenopteran insects is negatively affected by A3s/T3s whereas that of dipteran genes is negatively affected by G3s/C3s. Because the extent of codon bias increases with decrease in ENC values, negative effect of A/T on ENC in hymenopteran genomes suggests that codon bias in these insects is positively influenced by A/T composition in the 3^rd^ position of codons. On the other hand, G/C composition of 3^rd^ position of codon positively influences codon bias of genes of dipteran insects.

**Table 2:** Logistic regression coefficients of effective number of codons of genes within each genome as a function of base compositions at the 3rd position of codons.

Species	T3 (coefficient)	C3 (coefficient)	A3 (coefficient)	G3 (coefficient)
**Acep**	0.1646	0.31886	−0.16941*	0.17616
**Amel**	0.07496	0.42096	−0.45187*	0.09815
**Cflo**	−0.11056*	0.12552	−0.18964*	0.15015
**Hsal**	0.61619	0.46088	0.38442	0.57466
**Ihum**	0.15177	0.03946	−0.14279*	0.0167
**Nvit**	0.16536	0.16954	−0.08718*	0.10899
**Pbar**	0.16536	0.16954	−0.08718*	0.10899
**Aaeg**	0.021064	−0.499102*	0.395321	0.006531
**Agam**	0.26042	−0.4251*	0.61248	−0.31529*
**Cqui**	0.03675	−0.58019*	0.53595	−0.25906*
**Dana**	0.22246	−0.41474*	0.15414	0.16263
**Dere**	0.25741	−0.45443*	0.09742	0.05521
**Dgri**	0.33781	−0.19401*	0.07405	0.05251
**Dmel**	0.18168	−0.49788*	0.09567	0.08595
**Dmoj**	0.34224	−0.21943*	0.23972	0.08589
**Dper**	0.24326	−0.42259*	0.06055	−0.01846*
**Dpse**	0.25773	−0.45495*	0.11006	0.04144
**Dsec**	0.22148	−0.45229*	0.04583	0.03775
**Dsim**	0.25028	−0.42527*	0.1358	0.07849
**Dvir**	0.37258	−0.27864*	0.19328	0.03956
**Dwil**	0.33014	0.09225	0.21414	0.28882
**Dyak**	0.27863	−0.37124*	0.16749	0.09378

* p value of regression is significant (p<0.05).

### Amino Acid Composition Bias and its Association with Codon Usage Bias

The amino acid frequencies were calculated for each species in genome-wide manner (Table S2). Pair-wise comparison of amino acid frequencies between dipteran and hymenopteran species shows that they vary in highly correlated manner (Pearson correlation coefficient >0.93) between the two groups of insects. The leucine residues are present in highest frequency in the amino acid composition of proteins of each species (accounting for ∼9% of total amino acids) whereas tryptophan residues are least abundant (∼1%) in the proteins of each insect.

With a given gene, mutations in the codon sequences, particularly in the 1^st^ and 2^nd^ positions of codons, will determine the amino acid composition of the encoded protein of the gene. Thus, codon bias generated by such mutations can have an association with the observed bias in the composition of amino acids of the genome. We performed codon permutation analysis wherein the position of bases of codon sequences were randomized for all genes in the genome with an aim to test if observed data significantly deviated from the randomized data. The amino acid counts were determined in both observed and randomized datasets to calculate expected codon usages where the null assumption was that all synonymous codons are equally used to code each amino acid. The total counts of codons and the expected number of codons of the observed and the randomized data were used to test significance of association between codon bias and amino acid bias by 2×2 contingency analyses (Pearson’s Chi-squared tests). The results shown in Table S3 suggest that significant association exists between codon bias and amino acid bias for most of the codons in hymenopteran genomes but not in dipteran genomes. Only few specific codons in select dipteran genomes (particularly the mosquitoes) show such an association. The fruit fly species predominantly lacked this association. This indicates that codon bias associated with changes in amino acid composition (resulting from mutational bias in 1^st^ and 2^nd^ codon positions) is a characteristic feature of hymenopteran insects but not the dipteran insects. This doesn’t mean that mutations in the 1^st^ and 2^nd^ codon positions in dipteran genes are not associated with changes in amino acids. Rather, the statistical significance of these changes in codon bias and amino acid composition bias is distinct between the two groups of species. The distinction is most likely associated with mutational bias of 1^st^ and 2^nd^ codon positions of specific amino acids such as leucine, arginine and serine among the species (data not shown). Sequence changes in the 1^st^ and 2^nd^ position of codons of these amino acids can result in synonymous mutations unlike codons of other amino acids.

### Comparison of Synonymous Codon Usage Orders in Genome-wide Manner

We further analyzed synonymous codon usage orders (SCUO) of genes of each species. SCUO is a relatively newer approach compared to RSCU and is considered as more robust for comparative analysis of codon usage (see more in Methods). The SCUO analysis shows that a majority of the insect genes are associated with low codon usage bias. This is because the majority of genes (>72%) in each species have SCUO values less than 0.5 (SCUO = 0 for no bias and = 1 for highest bias of codon usage) (Table S4). Thus, genes with high codon bias are relatively low in number in each of these insects. Based on predicted gene ontology, genes with high codon bias seem to have association with specific functions such as translational processes, ribosomes (mostly ribosomal protein genes), intracellular activities, transport, oxidation-reduction process and others (Table 3).

**Table 3:** Top 20 gene functions of insect genes that are associated with biased synonymous codon usage orders (SCUO >0.5).

Rank	Predicted Function
**1**	Intracellular
**2**	Ribosome
**3**	Translation
**4**	Structural constituent of ribosome
**5**	Nucleus
**6**	DNA binding
**7**	Chromosome
**8**	Nucleosome
**9**	Nucleosome assembly
**10**	Structural constituent of cuticle
**11**	Ribonucleoprotein complex
**12**	Binding
**13**	Nucleotide binding
**14**	Integral to membrane
**15**	Membrane
**16**	Transport
**17**	Kinase activity
**18**	Protein binding
**19**	ATP binding
**20**	Oxidation-reduction process

**Figure 2:**
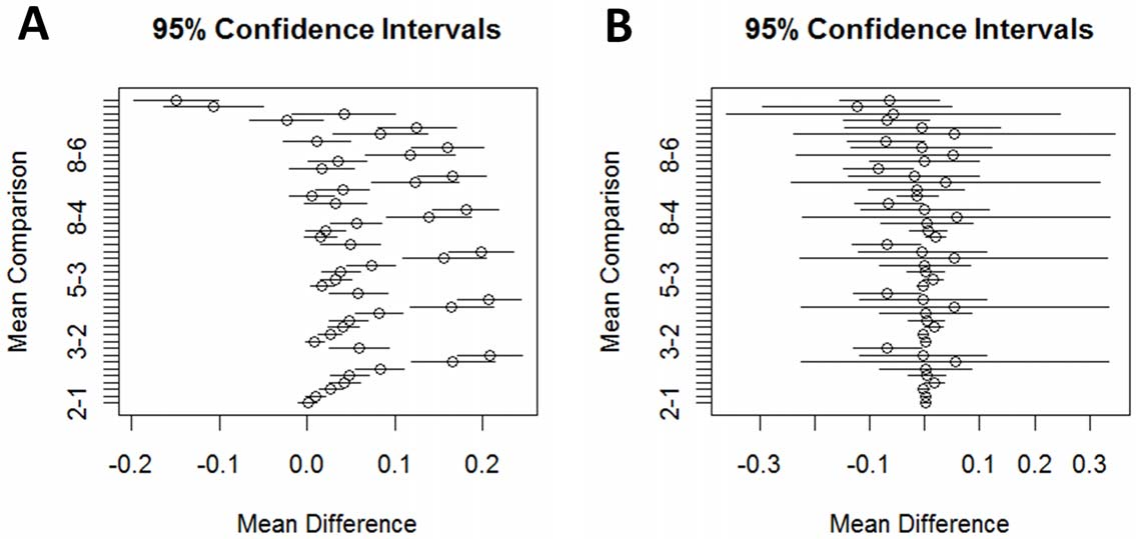
Representative SCUO variance plots. Mean differences of SCUO variance between gene groups with 10 different intervals (see Table S4) of codon usage orders. The pair-wise intervals are shown on y-axis and the mean differences are represented by the x-axis. The 95% confidence intervals for each of the pair-wise comparisons are shown by horizontal bars with the middle circle representing the mean difference value. The two plots represent two ant genomes; A) jumping ant (*Harpegnathos saltator*) and B) Argentine ant (*Linepithema humile*).

Furthermore, our data shows that synonymous codon usage orders of insect genes vary in species-specific manner. This is evident from pair-wise partitioning analysis of SCUO values of genes of each species. The genes in each species were partitioned into 10 non-overlapping groups with 0.1 intervals of SCUO values [*e.g*. 0–0.1, 0.1–0.2, …, 0.9–1.0]. The Dunnett’s modified method of Tukey-Kramer test (DKT) [19] was then used to perform pair-wise comparisons of codon usage variance among the 10 gene groups within each species (Figure 2). The results of these analyses suggested that codon usage variations are specific to each species. For example, the representative plots in Figure 2 show the variance of synonymous codon usage orders between two ant species, *H. saltator* and *L. humile*. It shows that the mean differences of variance of codon usages are very different between the two ants; they are low (near zero) in *L. humile* (Argentine ant) but are high in the *H. saltator* (jumping ant). We have performed these pair-wise analyses for each of the 22 species and the pattern of SCUO variation was different in each species (data not shown) clearly suggesting that codon bias patterns vary in species specific manner.

We also analyzed codon usage bias of common genes (n = 30) that were identified as one-to-one orthologs among 17 of the 22 species (orthology relationship obtained from Hierarchical Catalog of Eukaryotic Orthologs; http://cegg.unige.ch/orthodb4). Also, these 30 genes were chosen as they each had only one exon. Single exon genes have previously been shown as appropriate genes for codon bias comparison studies as introns are known to have effect on codon bias [20]. Based on the analysis of synonymous codon usage orders of these 30 genes, it was found that *Drosophila willistoni* had the least and *Apis mellifera* had the highest bias of codon usage orders among all the species (Table 4). Moreover, it was also found that the codon usage orders of the orthologous genes bear a significant correlation (Spearman rho = 0.808; p = 0.00004) with the genome-wide average SCUOs of the species. The correlated variation of SCUO bias between orthologous genes and all genes present in the genome (orthologous as well non-orthologous) further suggest that sampling of orthologous genes may be a reliable approach to predict codon bias among species.

**Table 4:** Average synonymous codon usage orders (SCUOs) of orthologous genes.

Species	Avr_ortho_	Avr_genome-wide_
Aaeg	0.115	0.148
Agam	0.3	0.218
Amel	0.411	0.272
Cqui	0.263	0.221
Dana	0.176	0.178
Dere	0.208	0.201
Dgri	0.153	0.162
Dmel	0.178	0.175
Dmoj	0.144	0.185
Dper	0.206	0.211
Dsec	0.22	0.205
Dsim	0.224	0.21
Dvir	0.174	0.177
Dwil	0.101	0.142
Dyak	0.216	0.205

The genome-wide averages of SCUOs are also shown.

### Patterns of Codon Context Variation among Species

**Figure 3:**
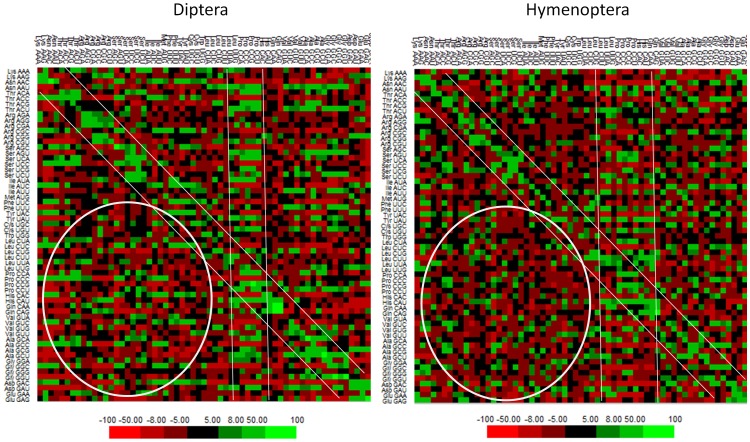
Patterns of codon context variation in dipteran and hymenopteran insects. The cluster pattern is based on average matrix of residuals of each codon context among the species of each order. The 5′ codons are in rows and the 3′ codons are in columns. The green color represents highest number of the contexts and red color represents the lowest number of contexts (scale is shown below the figure).

The codon context patterns of these species were investigated using the Anaconda software [13]. Results revealed that genome-wide frequencies of individual contexts are variable among species. The most frequent and the least frequent codon contexts of each species are listed in Table 5. We used the Anaconda software to determine the adjusted residual values for association of each codon pair in genome-wide manner for the 22 species (Table S5). The residual values signify the Chi-square test association between the two codons of each context [13]. Furthermore, based on average cluster patterns of adjusted residual values of codon pair frequencies among the species of Diptera and Hymenoptera, it was found that specific contexts were represented more often than other contexts. The cluster patterns revealed distinctions and as well as commonalities of codon context variations between Diptera and Hymenoptera (Figure 3). The regions shown by two parallel lines in Figure 3 indicate codon contexts that are relatively more frequent than other contexts among the species. The codon contexts localized diagonally from left top to right bottom in Figure 3 are also associated with higher frequency than other contexts. Being in the diagonal positions, they represent contexts of the same triplet sequences suggesting that these contexts (homogenous codon contexts) are generally frequent in these insects. Furthermore, codon context sequences NNN-CCA, NNN-CCC, NNN-CCG and NNN-CCU contexts (where N = A/T/C/G) were also observed as frequent contexts in both dipteran and hymenopteran insects (these are contexts of two different triplets or heterogeneous codon contexts). It was further found that, in most of these heterogeneous contexts, the 3′-codon predominantly codes for proline whereas the 5′codon is variable. In addition to such common features of context patterns between the two orders, differences in the context patterns were also observed as indicated by circles in Figure 3. They mostly correspond to the codon contexts that are relatively higher in frequency in dipteran species than the hymenopteran species.

**Table 5:** The most frequent and the most rare context sequences of codons in different species.

Species	Frequent context	Rare context
**Aaeg**	AAG-AAG (13906)	GGG-UAG (15)
**Acep**	GAA-GAA (11832)	CCC-UAG (9)
**Agam**	CAG-CAG (27317)	CCC-UAA (14)
**Amel**	GAA-GAA (3762)	ACC-UGA (1)
**Cflo**	GAA-GAA (13807)	GGG-UAG (9)
**Cqui**	CUG-CUG (22269)	GUA-UAG (17)
**Dana**	GAG-GAG (12924)	AGG-UAG (4)
**Dere**	CAG-CAG (30369)	CCU-UGA (18)
**Dgri**	CAG-CAG (36858)	CGG-UAG (12)
**Dmel**	CAG-CAG (61372)	GGG-UAG (12)
**Dmoj**	CAG-CAG (38644)	GGG-UAG (7)
**Dper**	CAG-CAG (31748)	GGG-UAA (22)
**Dpse**	CAG-CAG (38169)	CCG-UGA (15)
**Dsec**	CAG-CAG (25468)	GGG-UAG (8)
**Dsim**	CAG-CAG (22010)	GGG-UAG (12)
**Dvir**	CAG-CAG (36328)	GGG-UAG (8)
**Dwil**	CAG-CAG (21206)	CCG-UGA (9)
**Dyak**	CAG-CAG (29213)	GGG-UAG (11)
**Hsal**	GAG-GAG (12841)	CCC-UAG (8)
**Lhum**	GAA-GAA (9158)	CCC-UAG (9)
**Nvit**	GAA-GAA (15324)	GGG-UAG (12)
**Pbar**	GAA-GAA (8684)	CCC-UAG (5)
		

* Numbers in the parenthesis are the total counts of the codon context in the genome.

We then asked if the compositions of amino acid pairs in these insects are biased. The analysis shows that amino acid pairs are highly biased in each species (Table S6). For example, pairs of glutamic acid and proline are infrequent in each species compared to other amino acid pairs. On the other hand, presence of two glutamines as an amino acid pair is highly abundant in the proteins of these insects. The data in Table S6 shows that bias in amino acid pair composition is highly correlated (Pearson correlation coefficient >0.82) between dipteran and hymenopteran species. Thus, we took into consideration the amino acid pair frequencies in each species while comparing the codon context bias among the species. For this purpose we calculated ‘relative synonymous codon pair usage’ (RSCPU). The RSCPU is the observed frequency of codon pairs divided by the expected frequency which in turn is the total number of amino acid pairs divided by total number of codon pairs those code the same amino acid pair. The RSCPU values of individual codon pairs (60×60 pairs) for each of the 22 genomes are listed in Table S7. From this analysis, we identified specific codon pairs which are used more frequently than expected in dipteran insects compared to hymenopteran insects and *vice versa*. We found a total of 742 codon pairs which are more frequent in hymenopteran insects compared to dipteran insects. However, only 428 codon pairs show opposite trend: they are frequently used in dipteran insects compared to the hymenopteran insects.

### Codon Context Patterns Correspond to Species Phylogeny

**Figure 4:**
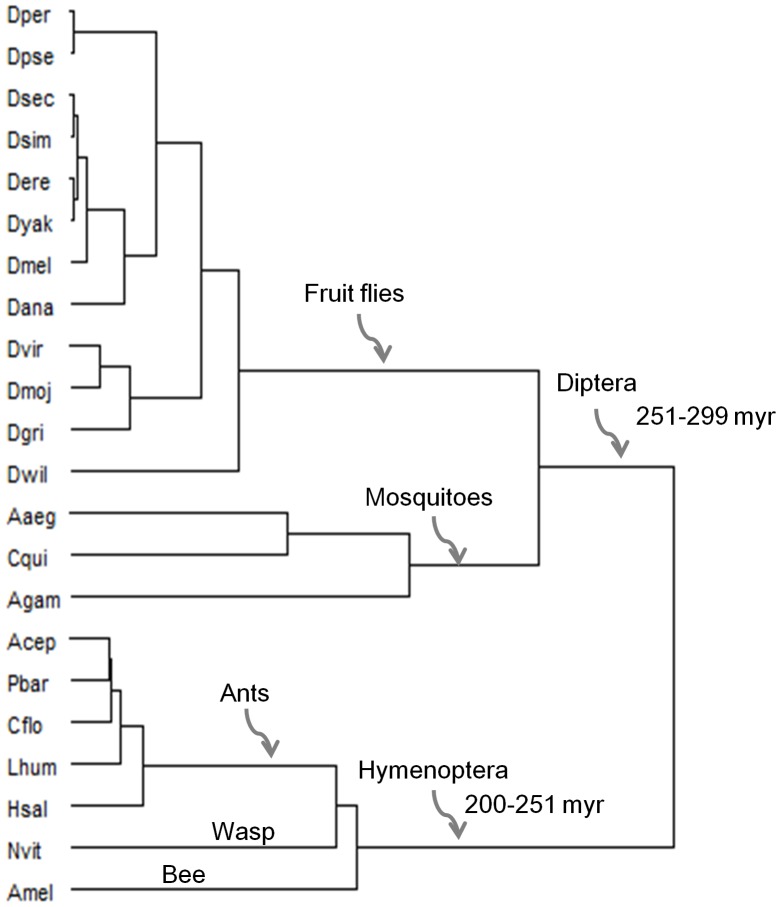
A cluster tree depicting codon context variation among insects. The species names are shown as 4 letter abbreviations. The clusters of codon context variation and their association with insect orders have been indicated. The divergence times (in million years from now) of Diptera and Hymenoptera orders are also shown.

We further observed that variation of codon contexts among insect genomes shows a good correspondence with the known phylogeny of the insect species (Figure 4). The cluster tree shown in Figure 4 represents the pattern of codon context variation among the species (note that it is not a phylogenetic tree). It shows that the variation of codon contexts among fruit flies is relatively more similar with that of mosquitoes than wasp, bee and ants. On the other hand; the ants, bee and wasp make a single cluster for codon contexts variation representing the hymenopteran phylogeny.

### Context Patterns of Start and Stop Codons

The codon contexts of the 5′-sequences of stop codons and 3′-sequences of the start codon were analyzed in these insects. The results of this analysis show that specific sequences are frequently used as contexts of start and stop codons of these insects. It was found that ‘A’ rich codons are frequent whereas C or G rich codons are generally avoided in the 5′-contexts of stop codons (Table 6). The triplet AAA (that codes for lysine) is the most frequent 5′- context of ‘ochre’ stop codon in many species. The ‘amber’ and ‘opal’ codons are associated with AAG as the frequent 5′-context in some species, mostly in the mosquitoes (Table 6). On the other hand, GGG, CCG and CCC codons are the most avoided 5′-contexts of stop codons of these insects. The 3′-codon context of AUG are highly variable among species (Table S8). However, it was found that codons starting with ‘GA’ are frequent whereas codons starting with ‘GG’ are generally avoided in the 3′-contexts of AUG. These results suggest that codon contexts of start and stop signal have certain degrees of non-random usage patterns in the genes of these insects.

**Table 6:** The most frequent (mf) and least frequent (lf) 5′- context sequences of stop codons UAA, UAG and UGA) in different species.

Species	(UAA)mf	(UAA)lf	(UAG)mf	(UAG)lf	(UGA)mf	(UGA)lf
**Aaeg**	AAG	CCC	AAG	GGG	AAG	CCC
**Acep**	AAA	CCC	AAA	CCC	AAA	ACC
**Agam**	AAG	CCC	AAG	GUA	AAG	GGG
**Amel**	AAA	CGC	AAA	AUC	AAA	ACC
**Cflo**	AAA	CGG	AAA	GGG	AAA	GGG
**Cqui**	AAG	GUA	AAG	GUA	AAG	CCC
**Dana**	AAA	CGG	AAG	AGG	AAG	GGG
**Dere**	AAG	CCG	AAG	GGG	AAG	CCU
**Dgri**	AAA	GGG	AAA	CGG	AAG	GGG
**Dmel**	AAG	GGG	AAG	GGG	AAG	CCG
**Dmoj**	AAA	GGG	AAA	GGG	AAG	CCG
**Dper**	AAA	GGG	AAG	CCU	AAG	CCG
**Dpse**	AAG	GGG	AAG	AGG	AAG	CCG
**Dsec**	AAA	GGG	AAG	GGG	AAG	CCU
**Dsim**	AAA	GGG	AAG	GGG	AAG	GGG
**Dvir**	AAA	GGG	AAA	GGG	AAA	CCG
**Dwil**	AAA	CCG	AAA	GGG	AAA	CCG
**Dyak**	AAG	GGG	AAG	GGG	AAG	CCG
**Hsal**	AAA	CCC	AAG	CCC	AAG	GGG
**Lhum**	AAA	GGG	AAG	CCC	AAA	CCC
**Nvit**	AAA	GGG	AAA	GGG	AAG	GGG
**Pbar**	AAA	CCC	AAA	CCC	AAA	GGC

## Discussion

The present investigation highlights the codon usage patterns in a comparative manner between two important insect orders, Diptera and Hymenoptera. The insect species analyzed in the study are important not only because they are relevant to transmission of human diseases (mosquitoes) or crucial to environment and agriculture (bees and wasp) but also for the fact that they include a model organism (fruit fly) for genetic research. As usage of synonymous codons during translation is non-random, identifying the patterns of codon usage bias is important towards understanding the mode of translational selection of protein coding genes among related species.

One of the observations from this study is that codons with ‘A’ at the 2^nd^ codon position are generally highly frequent whereas codons with G or U at the 2^nd^ position are avoided in most of these insects. This pattern of codon selection is most likely related to differential hydrophilicity or hydrophobicity of proteins. The universal nature of the genetic code is linked to the biochemical properties of amino acids [21]. It is known that most of the xAx (where x = any nucleotide) codons code for hydrophilic amino acids whereas xUx codons code for hydrophobic amino acids in nearly universal manner. Because ‘A’ at 2^nd^ position is maintained as optimal codon across these insect species, it is likely that selection of U over G at the 2^nd^ position of the avoided codons may influence hydrophilicity and hydrophobicity of the encoded proteins of these insects.

The results of hierarchical clustering of RSCU values of codons among the species (Figure 1) reveals that third positions of frequently used codons are mostly G or C in the dipteran insects. In the hymenopteran insects, the third positions of frequently used codons are mostly A or U. This is in agreement with earlier reports [10], [14] that also suggest frequent usage of G and C ending codons in the *Drosophila* species. In particular, CUG is the most frequent codon for leucine in *Drosophila* spp. as well as in mosquitoes (Table S1). On the other hand, AGA is the most frequent codon in the hymenopteran insects. The C or G ending codons in dipterans may be frequent due to higher overall GC content in *Drosophila* spp. and mosquito species compared to the hymenopteran insects [16] [22]. We also showed that codon bias was significantly associated with high GC content in dipterans but with low GC content in hymenopterans (Table 2). Furthermore, in *Drosophila* spp., higher numbers of genes show GC-biased selection as has been observed from comparisons of the codon adaptive index (CAI) with the effective numbers of codons (ENC) of genes among the 12 *Drosophila* species [14]. These results are in agreement with the results of our regression analyses (Table 2) suggesting that G3s/C3s tend to positively affect codon bias in dipteran species whereas A3s/T3s seem to affect codon bias in similar manner in hymenopteran species.

It should be noted that our analysis of codon bias and nucleotide composition bias is based on comparison between CAI and ENC. As CAI is a directional measure of codon bias unlike ENC, the CAI of genes dominated by mutation bias would have a lower values than those with completely even usage of codons. This has been the principle of ENC versus CAI comparison among the 12 *Drosophila* species where mutation bias is generally toward A/T while codon usage bias is generally toward G/C [14]. The results from our study although based on the same principle as that of [14], may have limited implications on relationship between codon bias and mutational bias as it is not clearly established that mutational events in all insect genomes are A/T biased. Although an earlier investigation [22] suggests that nucleotide composition of dipteran and hymenopteran genomes are very different, further studies are required to clarify if this difference may have significant influence on observed codon bias of genes between the two insect groups.

One of the limitations of the study is the lack of comparison of observed codon bias with gene expression data among the species. Our data shows that the majority of genes of these insect genomes tend to have SCUO within 0.1 to 0.2. The genes with higher codon usage order (with SCUO >0.5) are few in number in each species and represent specific gene functions. Generally, high codon usage bias is associated with highly expressed genes which in turn are genes that are least evolvable [6], [23]. In insects, the highly biased and highly expressed genes mostly represent house-keeping genes as evident from our earlier investigations [24], [25]. However, we have shown in an earlier work in mosquitoes that only 2–4% of genes of known biochemical pathways are associated with high codon bias which were also highly expressed [25]. Thus, lack of expression data has limited us to prove if the observed codon bias has any relation to gene expression and translation among the species. The other limitation of our analysis may be associated with gene annotation of these species. There are possibilities of sequence gaps and misannotation of genome sequences that can affect the quality of such analysis. Although evidence based (such as expression sequence tags or ESTs) gene annotation data is most appropriate for codon bias analysis, it is not however possible to have such data for all annotated genes from genome sequences.

Although several studies have clearly demonstrated the importance of codon contexts on structure and evolution of mRNA sequences [13] [26]–[27], no information is available on how codon context structures shapes the coding sequences of the insect species investigated in the current study. The results obtained from our analysis shows that codon pairs which are ubiquitously frequent across insect species are mostly homogenous (pairs of same codon triplets) (Figure 3). Usage of homogenous codon context may be energetically less expensive during translation than usage of contexts of different codon sequences corresponding to the A and P sites of ribosome. This is because, only one tRNA is synthesized that can carry out the aminoacylation of codons with homogenous contexts whereas two tRNAs are synthesized for heterogeneous codon contexts. Moreover, the role of tRNAs on codon context variation is known [28]. Also, over-utilization of a limited set of tRNAs may be difficult during translation, especially if the gene is highly expressed, due to reduction of available tRNAs that are correctly aminoacylated and modified. Also, it is reasonable to assume that there might be some constraints on codon repetitions. Codons cannot be repeated indefinitely or else proteins would have a very limited amino acid composition. The codon context analysis further reveals that specific codon sequences are used more often than others in the 3′ and 5′ contexts of the start codon and the stop codons, respectively (see Table 6 and Table S8). Biased usage of sequence contexts of start and stop codons may have a role on initiation and termination of translation of genes as has been suggested by other studies [29], [30].

The codon context patterns vary according to species phylogeny of the insects (Figure 4). Although co-evolutionary selection of the genetic code is known [31], the results indicate that codon context sequences may have an important association with species divergence. Furthermore, co-evolution of codon bias in insects can’t be ruled out when it is known that translational selection of *Drosophila* genes correspond to their phylogeny within the genus [15].

In conclusion, our study reveals that codon usage bias is an important feature of genome evolution in insects. The information from the study may help us better understand translational selection of insect genes. Although, complete genome sequences for several insect genomes have been sequenced, codon bias features have not been addressed in many of these genome studies. We suggest that analyzing codon structures from the forthcoming genome sequencing projects may provide valuable information about translational selection among related species.

## Materials and Methods

### Sequence Data and Sources

The 15 dipteran species analyzed in this study include twelve *Drosophila* species (*D. melanogaster*, *D. simulans*, *D. sechellia*, *D. yakuba*, *D. erecta*, *D. ananassae*, *D. pseudoobscura*, *D. persimilis*, *D. willistoni*, *D. grimshawi*, *D. virilis*, *D. mojavensis*] and three mosquito species [*Aedes aegypti* (*A. aegypti*), *Anopheles gambiae* (*A. gambiae*), *Culex quinquefasciatus* (*C. quinquefasciatus*)]. The seven hymenopteran insects studied include five ant species [leaf cutter ant (*Atta cephalotes*), carpenter ant (*Camponotus floridanus*), Argentine ant (*Linepithema humile*), jumping ant (*Harpegnathos saltator*) and red harvester ant (*Pogonomyrmex barbatus*)] and one species of wasp *Nasonia vitripennis* (*N. vitripennis*)] and honey bee [*Apis mellifera* (*A. mellifera*)]. The insect names have been abbreviated as the first letter of the genus followed by three letters of the species name throughout the text and illustrations. The annotated coding sequences (CDS) of the twelve *Drosophila* genes were obtained from FlyBase (www.flybase.org). They were CDS-r1.3 versions for each *Drosophila* species except *D. melanogaster* (r5.27), *D. pseudoobscura* (r2.10) and *D. virilis* (r1.2). The coding sequences of the three mosquitoes were obtained from Biomart (http://www.biomart.org). The CDS sequences of *A. mellifera* genes (pre-release 2), and the ant species (Acep OGS1.2, Cflo v3.3, Hsal v3.3, Lhum OGS1.2 and Pbar OGS 1.2) were obtained from http://hymenopteragenome.org/. The Nasonia (*N. vitripennis*) coding sequences (*N. vitripennis*_OGS_v1.2) were obtained from Baylor College of Medicine (http://www.hgsc.bcm.tmc.edu). The predicted protein sequences in each genome were also obtained from the respective sources.

### Codon Frequency and Relative Synonymous Codon Usage (RSCU) Index

The codon sequences of genes from each genome were obtained by aligning CDS sequences of genes with the respective protein sequences using ‘RevTrans’ software [32]. The codon sequences (5′–3′) were then subjected to the ‘Anaconda’ program [13] to count total number of codons for each species. The genes where start or stop codon have not been defined were excluded from the analysis. The relative synonymous codon usage (RSCU) value was calculated as described in [33]. Briefly, RSCU was determined by dividing the observed codon counts by the expected counts where the expected counts assumed random usage of the synonymous codons for each amino acid.

### Cluster Analysis

The RSCU values of codons among the 22 species were clustered using a hierarchical clustering method (average linkage) implemented in Cluster 3.0 software [34]. The rank order correlation based similarity matrix of the RSCU values was used to determine clusters among codons (columns) and species (rows). The clusters were viewed by the TreeView program (http://www.eisenlab.org/eisen/).

### Nucleotide Composition and Relationship with Codon Usage Bias

The GC content, nucleotide composition of silent positions, codon adaptation index (CAI) and the effective number of codons (ENC) of genes were determined by CodonW software (http://codonw.sourceforge.net/) in genome-wide manner for each species. The ribosomal protein genes of honey bee, mosquito and fruit fly [24], [25] were used as reference genes to calculate CAI of the hymenopteran species, three mosquito species and the 12 *Drosophila* species respectively. Scatter plots were generated between CAI and ENC values among genes for each species. Apart from comparison of CAI and ENC in each species, a set (n = 698) of evolutionary conserved genes across the species were also used to compare CAI and ENC between dipteran and hymenopteran species. The gene set was extracted from OrthoDB5 (http://cegg.unige.ch/orthodb5) as single copy orthologs across the phylogenies of Diptera and Hymenoptera. Pearson correlation was calculated between CAI and ENC values in case. The CAI value equal to 0.5 showed genes (distributed on both sides of CAI = 0.5 value) wherein CAI value either increased or decreased with increase of ENC values. To know the effect of base composition of third position of codons, we performed Poisson regression between ENC as the dependent variable and the A3s, T3s, C3s and G3s frequencies as the continuous independent variable. The generalized linear model to which the data was fitted was ENC ∼ A3s + G3s + C3s + T3s. All regression analyses were performed in *R*.

### Analysis of Amino Acid Composition

The total number of amino acids in each genome was obtained by using the Anaconda software [13] from which the frequency of each amino acid was calculated (total counts of the species amino acid divided by sum of counts of the 20 amino acids throughout the genome). Pearson correlation coefficients were determined for each pair-wise comparison between dipteran and hymenopteran species.

To know the relationship of amino acid composition with biased usage of synonymous codons, codon permutation analysis was performed. The randomization of bases of codon sequences is a useful approach to determine if an observed feature of an mRNA is associated with biased usage of synonymous codons [35]. We performed sequence randomization of codon sequences using ‘seqinr’ with the ‘position’ model. The randomized codon sequences were then used to estimate the total number of each codon and amino acid. The total numbers of amino acids were divided by the corresponding codon degeneracy of the amino acid to estimate the codon counts expected to code them if no codon usage bias existed. The total numbers of codons of these amino acids as well as the expected codon counts of the observed data were compared with the corresponding values of randomized data. Chi-square tests were conducted to determine significance of association between codon bias and amino acid bias.

### Quantification of Synonymous Codon Usage Orders (SCUO) of Genes

The codon usage bias of each gene was estimated using principles of ‘Shannon Information Theory’ [36]. The information theory has been used to describe entropy of codon sequences of genes by [37]. Subsequently, the synonymous codon usage order (SCUO) index was developed [38] based on the information theory. It is calculated as 1 minus the ratio of expected to observed entropy, where the expected value of the entropy assumes random usage of all synonymous codons of a given amino acid. Codon usage bias calculated by this approach is suitable for comparative analysis of codon usage bias within and between genomes [38]. The ‘CodonO’ software [39] was used to calculate the SCUO index of each gene in each of the 22 species. As SCUO values vary from 0 (no bias) to 1(most bias), we categorized low and high biased genes based on SCUO <0.5 and SCUO >0.5 respectively. The functional prediction of genes showing SCUO >0.5 were identified based on the annotated gene ontologies (GO) of the official gene sets (downloaded from www.biomart.org).

### Comparison of Codon Usage Orders within and between Genomes

While variation in GC percentage between species may influence codon bias across genomes, within-genome variation of codon bias can be influenced by the presence of GC-rich isochores [40]. The influence of isochores may persist beyond speciation events and, as a consequence, GC content of orthologs between species can be more similar than GC content of paralogs within species [41]. To better understand within genome variation of codon bias, we compared the SCUO values of genes within each genome to determine the variance of codon usage orders among genes. Genes in each species were grouped based on SCUO values from 0 (no bias) to 1 (maximal bias) with increment of 0.1. The Dunnett’s modified method of Tukey-Kramer test (DKT) [19] was used to perform pair-wise comparisons of codon usage variance among the 10 gene groups in each species. The DTK test is appropriate to compare codon bias as the group size varies from each other and variance of SCUO may not be similar among the groups.

We also compared codon usage orders of a subset of one-to-one orthologous genes (n = 30) among 17 insect genomes (http://cegg.unige.ch/orthodb4). Apart from being orthologous among species, these genes were also intron-free. For the comparative analysis, we chose to compare the intron-free genes because presence of introns is known to have confounding effects on codon bias [20]. The intron-free genes were identified from the official gene sets of each species based on the number of exons predicted in these genes (www.biomart.org).

### Codon Context Analysis

All codon context quantifications were performed using the Anaconda software [13]. The amino acid pairs and the residual values of each codon pair were also quantified from the coding sequences of each genome by the Anaconda program. The genomes were compared for codon context patterns by the same program to generate the cluster tree. The relative synonymous codon pair usage (RSCPU) values were calculated from the observed codon pair counts in each genome. The observed counts were divided by the expected counts to obtain the RSCPU value for each codon pair. The sum of numbers of all the codon pairs that code for the same amino acid pair was divided by the product of codon degeneracies of both the amino acids to obtain the expected values. As the expected values assumed that all codon pairs were equally likely to code the amino acid pair, the ratio of observed to expected value represented the measure of biased usage of specific codon pairs over other alternative pairs to code the same amino acid pair.

## Supporting Information

Figure S1 **Representative scatter plots between CAI and ENC of genes between species.** The upper panel shows comparison between CAI and ENC in *Anopheles gambiae* (Diptera) and *Nasonia vitripennis* (Hymenoptera) in genome-wide manner. The lower panel shows similar scatter plots between the two species of 698 genes which are single copy orthologous genes across the dipteran and hymenopteran sequenced genomes.
(TIF)

Table S1 **RSCU values of codons of each species.**
(XLSX)

Table S2 **Amino acid composition of each genome.**
(XLSX)

Table S3 **Association between codon bias and amino acid bias in different species.**
(XLSX)

Table S4 **Percentage of genes belonging to different SCUO groups in each species.**
(DOCX)

Table S5 **Residual values of codon pair association in different species.**
(XLSX)

Table S6 **Bias in the amino acid pair composition in insect genomes.**
(XLSX)

Table S7 **Codon pair composition relative to the amino acid pair composition in insect genome.**
(XLSX)

Table S8 **The most frequent and the least frequent 3′-context of AUG in different species.**
(DOCX)
