# Supplementary figures and images for: Comparative Analysis of Codon Usage Bias and Codon Context Patterns between Dipteran and Hymenopteran Sequenced Genomes
Source: PLoS One. 2012 Aug 17;7(8):e43111. doi: 10.1371/journal.pone.0043111 (PMC3422295; doi:10.1371/journal.pone.0043111)

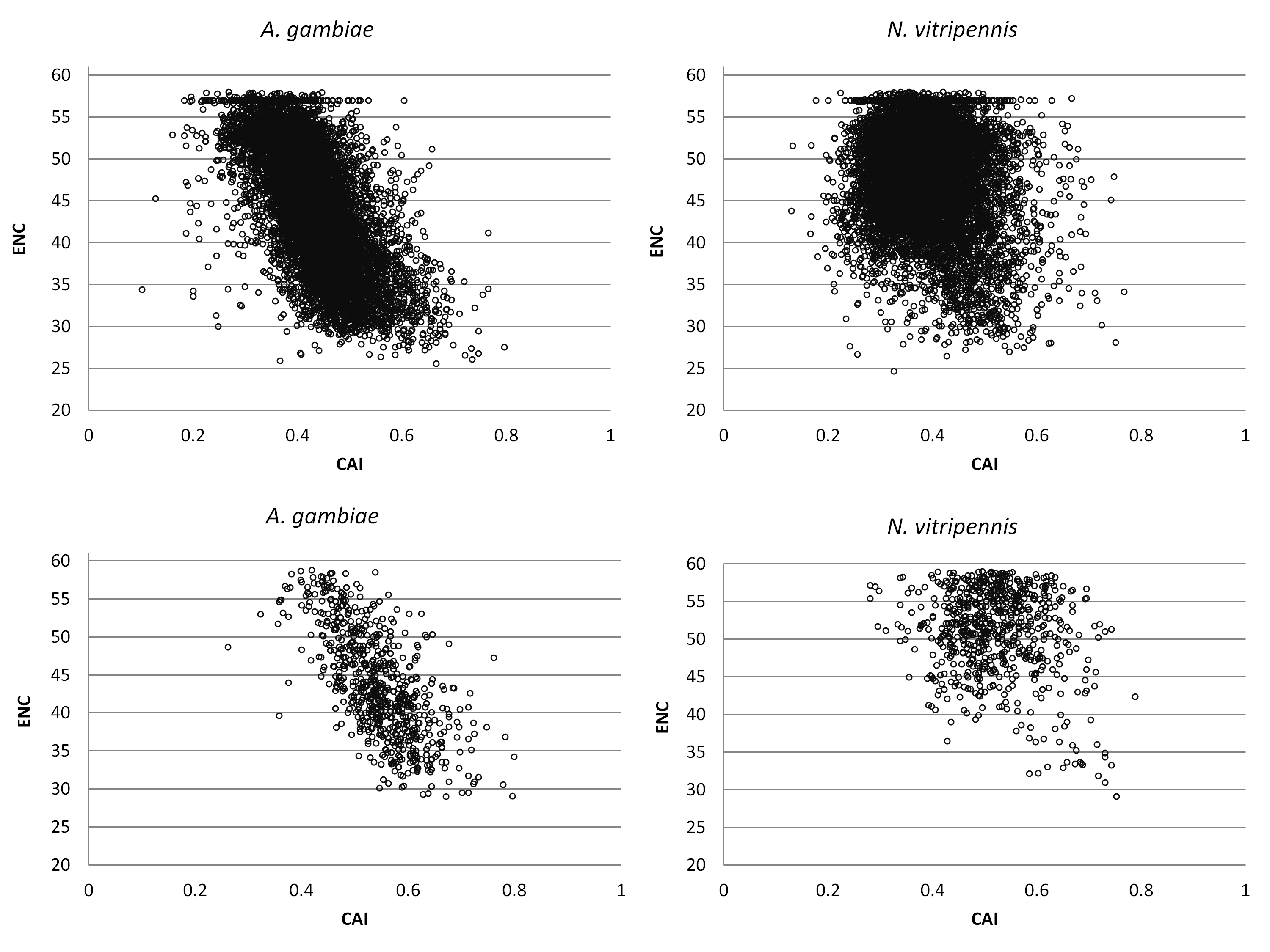

Supplement: Figure S1 — Representative scatter plots between CAI and ENC of genes between species. The upper panel shows comparison between CAI and ENC in Anopheles gambiae (Diptera) and Nasonia vitripennis (Hymenoptera) in genome-wide manner. The lower panel shows similar scatter plots between the two species of 698 genes which are single copy orthologous genes across the dipteran and hymenopteran sequenced genomes. (TIF) [file pone.0043111.s001.tif]
