# Supplementary material for: Comparative Analysis of Codon Usage Bias and Codon Context Patterns between Dipteran and Hymenopteran Sequenced Genomes
Source: PLoS One. 2012 Aug 17;7(8):e43111. doi: 10.1371/journal.pone.0043111 (PMC3422295; doi:10.1371/journal.pone.0043111)
Supplement: Table S4 — Percentage of genes belonging to different SCUO groups in each species. (DOCX) [file pone.0043111.s005.docx]

Table S4. Percentage of genes belonging to each SCUO group in each species. The percentage values are with respect to the total number of annotated protein coding genes in the species. The genes with SCUO within 0.1 to 0.2 constitute the highest proportion (indicated in red) of genes among the insect genomes.

| SUCO | 0-0.1 | 0.1-0.2 | 0.2-0.3 | 0.3-0.4 | 0.4-0.5 | 0.5-0.6 | 0.6-0.7 | 0.7-0.8 | 0.8-0.9 | 0.9-1 |
| --- | --- | --- | --- | --- | --- | --- | --- | --- | --- | --- |
| Aaeg | 39.59 | 38.12 | 13.42 | 5.65 | 2.33 | 0.74 | 0.12 | 0.03 | 0 | 0 |
| Acep | 27.16 | 37.04 | 21.42 | 10.06 | 3.2 | 0.72 | 0.25 | 0.06 | 0.04 | 0.04 |
| Agam | 15.95 | 35.07 | 26.43 | 13.94 | 6.21 | 1.95 | 0.39 | 0.05 | 0.01 | 0 |
| Amel | 16.25 | 21.08 | 18.19 | 21.66 | 17.21 | 4.55 | 0.77 | 0.24 | 0.03 | 0.02 |
| Cflo | 27.8 | 32.56 | 19.33 | 9.79 | 5.44 | 3.03 | 1.07 | 0.51 | 0.25 | 0.22 |
| Cqui | 13.4 | 37.43 | 28.07 | 12.56 | 5.15 | 2.31 | 0.65 | 0.21 | 0.14 | 0.08 |
| Dana | 26.27 | 41.85 | 18.47 | 7.99 | 3.54 | 1.51 | 0.29 | 0.08 | 0 | 0 |
| Dere | 19.96 | 38.54 | 23.18 | 10.23 | 5.91 | 1.88 | 0.25 | 0.04 | 0.04 | 0 |
| Dgri | 20.65 | 56.93 | 15.34 | 4.22 | 2.11 | 0.6 | 0.14 | 0 | 0 | 0 |
| Dmel | 25.44 | 42.69 | 20.19 | 6.97 | 3.49 | 0.86 | 0.18 | 0.18 | 0 | 0 |
| Dmoj | 21.36 | 44.05 | 21.17 | 9.11 | 2.5 | 1.13 | 0.34 | 0.15 | 0.13 | 0.07 |
| Dper | 13.66 | 40.75 | 26.93 | 11.72 | 4.93 | 1.48 | 0.38 | 0.12 | 0.01 | 0 |
| Dpse | 16.09 | 39.84 | 24.67 | 14.51 | 4.22 | 0.66 | 0 | 0 | 0 | 0 |
| Dsec | 18 | 38.55 | 24.25 | 11.71 | 5.2 | 1.66 | 0.48 | 0.11 | 0.03 | 0.01 |
| Dsim | 16.83 | 38.6 | 24.32 | 12.07 | 5.2 | 2.22 | 0.58 | 0.15 | 0.02 | 0 |
| Dvir | 19 | 49.85 | 20.54 | 7.52 | 2.32 | 0.49 | 0.08 | 0.1 | 0.06 | 0.04 |
| Dwil | 37.51 | 44.68 | 12 | 3.77 | 1.28 | 0.38 | 0.23 | 0.08 | 0 | 0.08 |
| Dyak | 18.88 | 38.33 | 23.67 | 11.19 | 5.21 | 2.03 | 0.48 | 0.13 | 0.06 | 0.02 |
| Hsal | 22.57 | 29.58 | 21.63 | 12.58 | 6.22 | 3.33 | 1.82 | 0.98 | 0.58 | 0.72 |
| Lhum | 32.08 | 36.68 | 19.3 | 8.3 | 2.66 | 0.65 | 0.24 | 0.03 | 0.03 | 0.03 |
| Nvit | 32.65 | 40.63 | 17.95 | 5.46 | 2.04 | 0.86 | 0.35 | 0.05 | 0.01 | 0.01 |
| Pbar | 29.32 | 35.29 | 19.97 | 9.92 | 3.58 | 1.16 | 0.49 | 0.15 | 0.07 | 0.06 |
