# Supplementary material for: Comparative Analysis of Codon Usage Bias and Codon Context Patterns between Dipteran and Hymenopteran Sequenced Genomes
Source: PLoS One. 2012 Aug 17;7(8):e43111. doi: 10.1371/journal.pone.0043111 (PMC3422295; doi:10.1371/journal.pone.0043111)
Supplement: Table S8 — The most frequent and the least frequent 3′-context of AUG in different species. (DOCX) [file pone.0043111.s009.docx]

Table S8. Sequences of the most frequent and the least frequent 3’-context of start codon (AUG) in different species.

| Species | Most preferred | Least preferred |
| --- | --- | --- |
| Aaeg | AUG-AAG | AUG-GGG |
| Acep | AUG-GAA | AUG-GGG |
| Agam | AUG-CUG | AUG-AGG |
| Amel | AUG-GAA | AUG-CGC |
| Cflo | AUG-GAA | AUG-GGG |
| Cqui | AUG-CUG | AUG-GGG |
| Dana | AUG-GAG | AUG-UUA |
| Dere | AUG-GAG | AUG-UUA |
| Dgri | AUG-CUG | AUG-UUA |
| Dmel | AUG-GAG | AUG-GGG |
| Dmoj | AUG-CUG | AUG-GGG |
| Dper | AUG-GAG | AUG-AGG |
| Dpse | AUG-GAG | AUG-UUA |
| Dsec | AUG-GAG | AUG-UUA |
| Dsim | AUG-GAG | AUG-UUA |
| Dvir | AUG-CUG | AUG-UUA |
| Dwil | AUG-GAU | AUG-AGG |
| Dyak | AUG-GAG | AUG-GGG |
| Hsal | AUG-GAG | AUG-UUA |
| Lhum | AUG-GAA | AUG-GGG |
| Nvit | AUG-GAA | AUG-GGG |
| Pbar | AUG-GAA | AUG-GGG |
